# Potential for a Combined Oral Inactivated Whole-Cell Vaccine Against ETEC and *Shigella*: Preclinical Studies Supporting Feasibility

**DOI:** 10.3390/vaccines13050513

**Published:** 2025-05-13

**Authors:** Manuela Terrinoni, Jan Holmgren, Kevin Ross Turbyfill, Lillian Van De Verg, Nicole Maier, Richard Walker

**Affiliations:** 1 Department of Microbiology and Immunology, University of Gothenburg, 40530 Gothenburg, Sweden; jan.holmgren@microbio.gu.se; 2 Walter Reed Army Institute of Research, Silver Spring, MD 20910, USA; kevin.r.turbyfill.civ@health.mil; 3 Center for Vaccine Innovation and Access, PATH, Washington, DC 20001, USA; lillian.verg@pathforbusiness.com (L.V.D.V.); nmaier@path.org (N.M.); rwalker@path.org (R.W.)

**Keywords:** combined vaccine, oral vaccine, ETEC vaccine, *Shigella* vaccine, dmLT adjuvant, mucosal immunity

## Abstract

Background: Enteric disease caused by *Shigella*, *Campylobacter*, and enterotoxigenic *Escherichia coli* (ETEC) represents a significant global health burden, particularly among children in low-resource settings. However, no licensed vaccines are currently available for these bacterial pathogens. Given the wide range of enteric pathogens and the constraints posed by an increasingly crowded infant immunization schedule, the development of combination vaccines or combined administration of individual oral vaccines may offer a practical approach to address this unmet need. Objectives: In this study, we evaluated the combined administration of two multicomponent oral vaccine candidates: ETVAX, targeting ETEC, and a trivalent whole-cell vaccine targeting *Shigella*. Methods: The vaccine candidates were administered orally in mice, both individually and in combination, with and without the inclusion of the double-mutant heat-labile toxin (dmLT) adjuvant. Results: The results demonstrated systemic and intestinal-mucosal immune responses to the key protective antigens following both individual and combined vaccine administration. Importantly, the combination of the two vaccines did not compromise the elicitation of specific antibody responses. The inclusion of dmLT as an adjuvant significantly enhanced immune responses to several antigens, highlighting its potential to improve vaccine efficacy. Conclusions: These findings underscore the feasibility of combining ETEC and *Shigella* vaccine candidates into a single formulation without compromising immunogenicity. This combined approach has the potential to provide broad protective coverage, thereby mitigating the global impact of enteric diseases and streamlining vaccine delivery within existing childhood immunization programs. Our results support further development of this combination vaccine strategy as a promising tool in combating enteric infections and improving health outcomes, particularly among young children in endemic regions who are vulnerable to enteric disease.

## 1. Introduction

Enteric disease caused by *Shigella*, *Campylobacter*, and enterotoxigenic *Escherichia coli* (ETEC) accounts for at least one-third of the diarrhea deaths in children and a similar proportion of the total cases in all age groups in low- and middle-income countries (LMICs) [1,2,3]. In addition, these bacterial enteric pathogens remain important causes of diarrhea and dysentery among international travelers and military personnel deploying to endemic areas [4,5,6,7]. There is no licensed vaccine against any of these bacterial enteric pathogens. Despite decreasing overall enteric disease mortality rates, approximately 525,000 deaths still occur annually, among which more than 400,000 are in children below age 5 y, and the total number of enteric infections (1.7 billion) each year remains high [8,9]. Rapidly progressing amplifiers, such as climate change, are also increasing cases of enteric disease [10,11,12]. In young children, repeated episodes of diarrhea due to enteric infections can also cause stunting and cognitive defects, which impact negatively on overall health and economic productivity during the life course, as well as increased susceptibility to other diseases [13,14,15]. Further, enteric pathogens are rapidly gaining antimicrobial resistance (AMR), pointing to the need for urgent action to prevent the climbing death toll and enteric disease burden associated with AMR organisms [16,17].

Due to the number of pathogens capable of causing enteric diseases and the increasingly crowded infant immunization schedule, the development and introduction of combined or co-administered enteric vaccines is important to address this major health threat [18]. These options may not only better address the polymicrobial or syndromic nature of both AMR and stunting than standalone individual vaccines, but they could also enhance uptake and public health impact [4,15,16,18,19,20,21]. Orally administered multi-pathogen vaccine strategies with combined or co-administered vaccines could offer an effective way to provide the broad coverage needed to impact enteric diseases [16,19,20]. Inactivated bacterial cells engineered to be maximally immunogenic and broadly cover specific groups of pathogens should be considered as an option for vaccine development. In the future, inactivated whole cells could be induced to overexpress key antigens, with particular focus on conserved protective antigens to maximize coverage, including antigens of heterologous pathogens to both further broaden coverage and simplify formulations [22].

Considerable progress towards the development of oral vaccines to protect against enteric pathogens has already been made. Several inactivated whole-cell, oral cholera vaccines are on the market, prequalified by the World Health Organization and used both for preemptive vaccination and epidemic control [23]. Other bacterial inactivated whole-cell, oral vaccine candidates (e.g., against ETEC and *Shigella*) are under clinical development and testing. Further, the mucosal adjuvant double-mutant heat-labile toxin (dmLT) [24,25] has been used in numerous vaccine studies to improve both systemic and mucosal immune responses against enteric pathogens, including to the most advanced ETEC vaccine candidate, ETVAX.

ETVAX contains orally administered, inactivated bacterial cells of four strains of ETEC expressing the most prevalent adhesin antigens for broad coverage, as well as a cholera-ETEC hybrid labile toxin B-subunit (LCTBA) and dmLT. ETVAX has completed Phase 2 trials in both adult travelers and children in LMICs with encouraging efficacy results [19,26,27,28,29,30,31,32]. In addition to inducing protective antibodies against wild-type, virulent heat-labile toxin, dmLT is dose-sparing and can significantly improve the immune responses to oral vaccines such as ETVAX in children who are known to be less responsive to oral vaccines than adults [33,34]. Initial field trial results indicate that ETVAX can provide encouraging levels of efficacy against ETEC-associated diarrhea in both adult travelers and LMIC infants [19,27,29,31]. Earlier studies of a predecessor to ETVAX in travelers showed that subjects mounting stronger than median serum IgA to the (cholera toxin) B subunit component of the vaccine candidate had a reduced risk of infection not only with ETEC strains known to share an antigen (LT toxin and/or colonization factor) with the vaccine candidate, but also against other enteric pathogens, like *Campylobacter* and *Salmonella*, which share antigens with the vaccine candidate, although this result is less well established [34,35,36,37]. Vaccine candidate responders also had a reduced risk of developing moderate-to-severe travelers’ diarrhea, independent of etiology [38]. These initial observations warrant further investigation of ETVAX’s potential as a platform for combined or co-administered oral enteric vaccine development.

An important step to further reduce the impact of bacterial diarrhea and to help accelerate combination vaccine development will be to show that a *Shigella* vaccine component consisting of a minimum number of serotypes and species can achieve broad coverage against shigellosis. An oral, inactivated *Shigella* whole-cell vaccine candidate has been shown to protect animals, and orally administered monovalent prototypes of either inactivated whole cells of *S. sonnei* or *S. flexneri* 2a have been safe and immunogenic in human volunteers [39,40,41].

Based on the promise of these two oral vaccine approaches, we conducted a preclinical study to see if a trivalent (*S. flexneri* 2a and 3a, and *S. sonnei*) *Shigella* whole-cell candidate could be combined effectively with ETVAX and retain balanced safety and immunogenicity. Immune interference, which is the relative enhancement or suppression of immune responses when vaccines are given at the same time as compared to separately, is a key concern. Interference may be related either to the vaccine formulation or to vaccine antigens and the immune response they elicit. This effect may arise due to antigenic competition, variations in immunodominance, or adjuvant-mediated modulation of immune pathways. Indeed, these challenges might be even more demanding for vaccines whose protective efficacy depends on their ability to induce an effective mucosal immune response as the mucosal immune system is more tightly regulated than the systemic immune system, which is mainly targeted by parenteral vaccines.

## 2. Materials and Methods

### 2.1. Vaccines and Adjuvant

A prototype of the multicomponent ETEC vaccine candidate ETVAX [42] containing all the components of the final ETVAX product except the dmLT adjuvant was obtained from Scandinavian Biopharma, Stockholm (SB). Each human dose vial contained an estimated total number of 1 × 10^11^ formalin- or phenol-inactivated *E. coli* bacteria together with 1.0 mg LCTBA B-subunit protein in 14 mL total volume (OD_600nm_ = 5.0), with the bacterial content being equally distributed on four inactivated engineered *E. coli* strains having defined surface-expressed amounts of CFA/I, CS3, CS5, and CS6 antigens, respectively, and except for the CS6 strain being of O78 serogroup.

A trivalent *Shigella* vaccine candidate, composed of a mixture of equal numbers of formalin-killed bacteria of the three strains *S. flexneri* 2a, *S. flexneri* 3a, and *S*. *sonnei*, in total 7.5 × 10^10^ bacteria per human dose, was developed at the Walter Reed Army Institute of Research (WRAIR) [43]. The inactivated vaccine bulks, each at OD = 50, were prepared at WRAIR and provided by PATH as individual components that were mixed in equal proportions for the study.

Purified dmLT adjuvant protein [25] was prepared under Good Manufacturing Practice (GMP) at the WRAIR Pilot Bioproduction Facility, Silver Spring, MD, USA, and kindly provided by SB; a human oral dose is estimated to be in the order of 10–50 µg.

### 2.2. Antigens

Purified ETEC colonization factor antigens CFA/I, CS3, CS5, and CS6; purified LPS O78 antigen; and LTB protein antigen were prepared as described [42]. *S. flexneri* 2a LPS, *S. flexneri* 3a LPS, and *S. sonnei* LPS and IpaB, IpaC, and IpaD antigens were prepared as described [43] and provided by PATH.

### 2.3. Immunizations and Collection of Samples

#### Mice

Six- to eight-week-old female F1-hybrid (CB6F1/OlaHsd) mice, purchased from ENVIGO, The Netherlands, Horst were housed under specific-pathogen-free conditions. All treatments and procedures were performed in accordance with the Swedish Animal Welfare Act (1988:534) and the Animal Welfare Ordinance (1988:539). The study was approved by the Ethical Committee for Laboratory Animals in Gothenburg, Sweden (Ethical permit number 1456/13).

Humane endpoints were used to minimize animal suffering. Mice were monitored daily for the first three days after immunization for visual signs of adverse reactions (reduced activity or responsiveness, affected fur coat condition and/or body posture) and then at least three days a week during each experiment, and body weight was recorded just before and 2 days after each completed round of administration. Animals were sacrificed if they had a weight loss of more than 10% or showed signs of apathy or loss of fur.

### 2.4. Immunizations and Collections of Specimens

**Figure 1:**
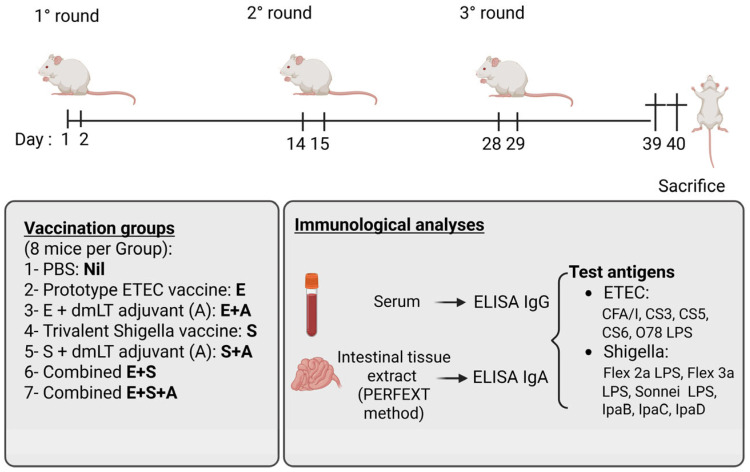
Summary outline of immunization groups and schedules, and of collection of test samples and analytic procedures.

Immunization groups, vaccination schedules, sample collection and analytic procedures are summarized in Figure 1. Eight mice per group were immunized orally by intragastric gavage in three rounds at two-week intervals. Each round comprised two administrations on consecutive days of 250 µL of vaccine preparation. Each administration dosage corresponded to 1/100th of an estimated human dose of ETVAX (“E”, ca 1 × 10^9^ bacteria and 10 microgram B subunit), trivalent *Shigella* vaccine (“S”, ca 7.5 × 10^8^ bacteria) or the combined E + S, together with or without 15 micrograms of dmLT adjuvant. The vaccine dosages had, in pre-tests, been found to be the highest possible to avoid significant adverse reactions when giving combined E + S (±A) vaccines. After mixing with 100 µL 6% (*w*/*v*) sodium bicarbonate buffer, the vaccine preparations were given to the animals perorally through a disposable feeding needle with a silicon tip (Fuchigama Ltd., Kyoto, Japan). A similarly sized control group (Nil) given only PBS with bicarbonate was also included.

Eleven days after the last vaccine administration, the animals were anesthetized with isofluoran and bled to death through cutting the pulmonary aorta. Serum was prepared by removing cells from blood samples by centrifugation and was stored at −20 °C until analyzed. Directly after bleeding, intestinal tissue was obtained from the sacrificed mice by the PERFEXT method as detailed elsewhere [44,45]. In short, the mice were extensively perfused intravenously with a heparin–PBS solution to remove remaining blood from tissue, followed by excision of a ca 3 cm long segment of the uppermost small intestine, which was stored at −20 °C in a protease inhibitory solution (1 mL per gram of tissue). Before analysis, the sample was thawed, ice-cold saponin (Sigma, St. Gallen, Switzerland) was added to permeabilize cells, and after centrifugation, the supernatant (referred to as upper intestinal tissue extract) was analyzed for antibody content by ELISA.

### 2.5. Immunological Analyses

The serum and upper small intestine tissue extracts were analyzed for IgG and IgA antibodies, respectively, to the ETEC CF antigens CFA/I, CS3,CS5, CS6; to LPS O78; to CTB; to *Shigella* LPS antigens from *S. flexneri* 2a, *S. flexneri* 3a, and *S. sonnei*; as well as to IpaB, IpaC, and IpaD antigens; for these analyses, the ELISA method was used as described previously [42,43], except for the antigen coating of the plates. For the latter, high-binding ELISA plates (Greiner) were coated overnight at 4 °C with 100 microliters/well of antigen in concentrations that pre-tests had identified as optimal: 1.5 µg/mL of CFA/I 1.5 µg/mL, 2.5 µg/mL of CS3, 4.5 µg/mL of CS5, 4.5 µg/mL of CS6, 5 µg/mL of O78 LPS, 5 µg/mL of *Shigella flexneri* 2a, 5 µg/mL of *Shigella flexneri* 3a, 5 µg/mL of *Shigella sonnei,* and 1 µg/mL of IpaB or C or D. ETEC antigens were diluted in PBS (Phosphate-Buffered Saline), whereas the *Shigella* antigens were in a carbonate buffer. Endpoint titers are expressed as the dilution of serum that gave an absorbance at 490 nm of 0.4 above background, calculated using Gene5 software (BioTek, Solna, Sweden). Mice in the vaccine groups having responded with a significant antibody titer rise to the different vaccine antigens were defined as those with an antibody titer exceeding the mean + 2SD antibody titer level for the Nil control group.

### 2.6. Statistical Analysis

The Prism software system GraphPad 10 (GraphPad Software, Inc., San Diego, CA, USA) was used for the statistical analyses. One-way ANOVA with Holm–Šidák post-test correction for multiple comparisons was used for analyses of differences between groups.

## 3. Results

Using intragastric gavage administration, we immunized mice in three rounds at biweekly intervals with either or both ETVAX and the trivalent *Shigella* vaccine candidate, and with or without the dmLT adjuvant. The serum IgG and intestinal-mucosal IgA antibody responses induced were measured after the last immunization to assess the following: (i) the responses to the key protective antigens after immunization with each vaccine candidate alone; (ii) whether the combined administration of these vaccine candidates would impact on the immunogenicity; and (iii) whether the mucosal adjuvant dmLT, when co-administered with the vaccine candidates individually or in combination, would enhance immune responses without compromising safety.

**Figure 2:**
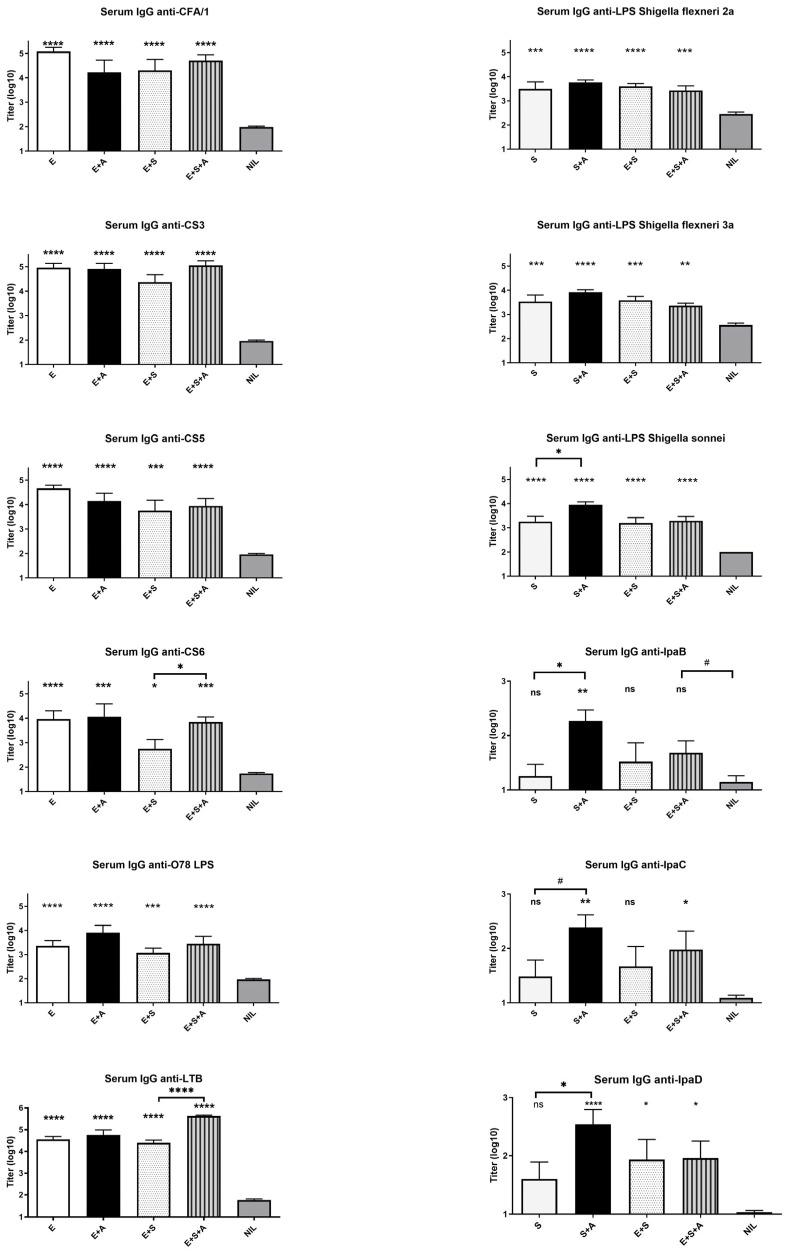
Serum antibody responses to different ETEC and *Shigella* antigens after three oral rounds of immunization with tetravalent whole cell + LCTBA B-subunit ETEC (ETVAX, E) vaccine and trivalent *Shigella* vaccine (S) alone or combined and administered with or without dmLT adjuvant (A). An unvaccinated group of mice served as negative controls (Nil). Statistical significances between antibody titers in immunized versus control mice were analyzed by one-way Anova with the Holm–Šidák correction for multiple comparisons, with *p* values indicated as * *p* < 0.05, ** *p* < 0.01, *** *p* < 0.001 and **** *p* < 0.0001 or ns = *p* > 0.05 on top of the bars. Statistical differences between antibody responses of selected vaccine groups were analyzed with the same method and are indicated by the horizontal lines linking adjacent bars; significant *p* values are indicated by the same symbols and insignificant trends as # *p* = 0.05–0.10.

**Serum antibody responses.** In the dosages used, none of the vaccines gave rise to any noticeable visual signs of adverse reactions or significant loss in body weight after administration or deviations in body weight gain during the course of the experiment compared to the control group given only buffer. Figure 2 shows the serum antibody responses to the different ETEC and *Shigella* vaccine antigens. Significant IgG antibody responses were observed in all immunization groups against all examined antigens as compared to the antibody levels in the unvaccinated control animals. However, although the frequency of mice responding with a significant antibody titer rise (defined as a titer exceeding the mean + 2SD titer for the Nil control group) was high at 75–100%), for all ETEC antigens, as well as for the three *Shigella* LPS antigens, the antibody responses evoked by the *Shigella* vaccine candidate were weaker than those induced by the ETVAX prototype vaccine, resulting in approximately 10-fold titer rises for *Shigella* as compared to 100- to 1000-fold titer rises for ETEC; for the anti-Ipa antigen responses, this was also reflected in low responder frequencies of 25–50% in groups not receiving the dmLT adjuvant. Importantly, combining the ETEC and *Shigella* vaccines, with or without dmLT, overall did not negatively affect the antibody responses to either vaccine candidate, especially not when the combined vaccine candidates were given together with dmLT.

The addition of dmLT in combination with S + E did not confer any noticeable adverse reactions, but as seen in the left panel of Figure 2, did significantly enhance the IgG response to LTB but not to the other ETEC antigens (although there was a tendency to enhanced antibody responses also to CS6 and O78 LPS). Regarding the *Shigella* serum IgG antibody responses, the addition of dmLT (S + A) significantly enhanced responses induced by the *Shigella* vaccine candidate against the S. *sonnei* LPS antigen, as well as against the Ipa protein antigens (IpaB, and IpaD) (Figure 2, right panel). The antibody responses to the other two *Shigella* LPS antigens, *S. flexneri* 2a and *S. flexneri* 3a, and to the IpaC protein, also tended to be enhanced by the addition of the dmLT adjuvant. For the IpaC and IpaD antigens, the addition of dmLT also increased the frequencies of significant responses to 88–100%.

**Intestinal-mucosal antibody responses.** Mucosal IgA responses were measured in duodenal-upper jejunal tissue sample extracts collected and prepared using the PERFEXT method which ensures that IgA antibody titers measured to ≥95% represent locally produced mucosal antibodies.

**Figure 3:**
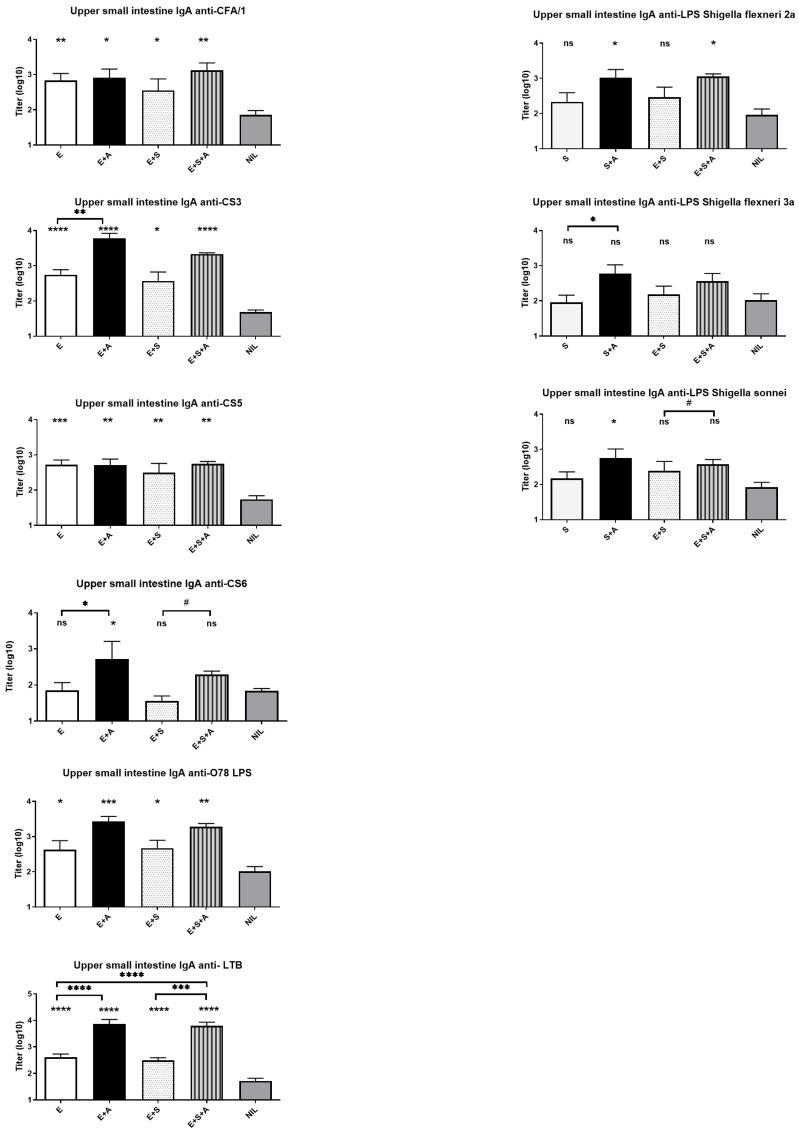
Intestinal-mucosal antibody responses to different ETEC and *Shigella* antigens after three oral rounds of immunization with tetravalent whole cell + LCTBA B-subunit ETEC (ETVAX, E) vaccine and trivalent *Shigella* vaccine (S) alone or combined and administered with or without dmLT adjuvant (A). An unvaccinated group of mice served as negative controls (Nil). Horizontal lines linking bars indicate significant differences between antibody responses of selected vaccine groups, as analyzed with the same method, with significant *p* values indicated by the same symbols and insignificant trends as # *p* = 0.05–0.10. * *p* < 0.05, ** *p* < 0.01, *** *p* < 0.001 and **** *p* < 0.0001 or ns = *p* > 0.05.

The results are shown in Figure 3. Significant IgA antibody responses were detected against all ETVAX antigens. Consistent with the serum antibody results, no negative impact on immunogenicity was observed from the co-administration of the ETEC and *Shigella* vaccines, whether dmLT was included or not. However, responses to the different *Shigella* LPS antigens were lower compared to those against ETEC O78 LPS and several of the ETEC CF antigens, and no responses against the Ipa antigens were detected above the levels seen in the Nil control group. Notably, the anti-*Shigella* LPS responses were significantly elevated only in groups that received the dmLT adjuvant. The adjuvant also tended to enhance the antibody responses to the *E. coli* O78 LPS antigen. Further, the intestinal IgA responses to the ETEC LTB antigen and to CF antigens, specifically CS3 and CS6, were enhanced by the co-administered dmLT adjuvant.

## 4. Discussion

Preclinical studies in mice were used to show that an inactivated *Shigella* whole-cell vaccine candidate consisting of three different cell types plus the four cellular components of ETVAX could be combined safely and with no apparent detrimental effect on the development of either anti-*Shigella* or anti-ETEC immune responses. The serum IgG and intestinal-mucosal IgA antibody responses induced were unaltered when measured after the last immunization. These data demonstrated that the responses to the key protective antigens after immunization with each vaccine alone and in the combined administration of these vaccines had no negative impact on their immunogenicity.

However, even when not combined, responses to the different *Shigella* LPS antigens were lower compared to those against ETEC O78 LPS and several of the ETEC CF antigens. This could be associated with unique biological characteristics of the two pathogens or, more likely, due to the way in which the two cellular vaccines were prepared. ETVAX consists of *E. coli* cells engineered to hyper-express the selected ETEC antigens [19,28,30,34]. In contrast, no manipulation of antigen expression was performed on the *Shigella* cells. Despite these differences in immunogenicity of the two cell types, the immune responses to the cells were unaffected on combination, further suggesting the potential for combination of multiple cells in the development of enteric whole-cell vaccines. In addition, the *Shigella* vaccine component(s) could also be induced to better express key antigens in future studies.

The mucosal adjuvant dmLT, when co-administered with the vaccines individually or in combination, enhanced immune responses without noticeably compromising safety. After the addition of dmLT, the *Shigella* vaccine induced significantly enhanced *Shigella* serum IgG antibody responses against the *S. sonnei* LPS antigen as well as against the Ipa protein antigens (IpaB and IpaD). Also, the antibody responses to the two other *Shigella LPS* antigens, *S. flexneri* 2a and *S. flexneri* 3a, as well as against IpaC, tended to be enhanced by the addition of the dmLT adjuvant. The adjuvant also tended to enhance the antibody responses to the *E. coli* O78 LPS antigen. Such adjuvant enhancement of LPS responses to the O78 component in ETVAX as well as of the intestinal IgA responses to ETEC LTB antigen and to CF antigens, specifically CS3 and CS6, has been seen in human trials and offers further evidence that this adjuvant can improve immune responses to both protein and polysaccharide antigens [34].

These study findings show that up to seven cell types could be combined without any loss in immunogenicity and that dmLT could enhance the immune responses to both pathogens. It should be remembered that ETVAX, which has successfully completed Phase 2 trials in both travelers and in infants and children in LMICs, is formulated to contain dmLT as well as a hybrid cholera B subunit-ETEC subunit, termed LCTBA, with a buffer [19,28,30,31,34]. Thus, ETVAX could provide adjuvant and antigen benefit to cells of other bacterial species as it did for the trivalent *Shigella* vaccine candidate used in these studies.

It should be noted that in the mouse studies, the two vaccine candidates were combined before oral administration. An alternative, more practical approach for orally administered vaccines than for parenterally injected vaccines is that, instead of combination, a cellular vaccine, such as that for *Shigella*, could be co-administered in association, but not physically combined as a product, with a vaccine like ETVAX, which contains buffer and dmLT, which could still benefit both vaccines. This possibility remains to be directly tested.

Although at least seven cell types were successfully combined in these studies, for practical vaccine development, every effort should be made to minimize the number of cell types included. A recently described *Shigella* Truncated Mutant (STM) could provide broad coverage with a single cell instead of three as used in our studies [46]. The STM is novel in that it involves the unmasking by genetic manipulation of conserved surface antigens on *S. flexneri* 2a cells by limiting the length of O-polysaccharide chains synthesized to one repeating unit. This broadens protective immunogenicity among *Shigella* species by better exposing surface proteins, such as conserved and protective Type-3 secretion system *Shigella* antigens. Inactivated STM cells combined with dmLT as an adjuvant induced potent antibody responses to the outer membrane proteins of the pathogen, eliciting cross-protective immunity against *S. flexneri* 2a, *S. flexneri* 3a, *S. flexneri* 6, and *S. sonnei* in a mouse pneumonia model [46]. Thus, *S. flexneri* 2a STM represents a promising candidate strain for a universal *Shigella* vaccine that could be compatible with combination or co-administration in conjunction with ETVAX or other oral vaccines. This novel approach is also gaining support from stakeholders in LMICs given the crowded childhood immunization landscape [47].

It is also possible to express heterologous conserved antigens on a cell type in the vaccine to achieve broader coverage without increasing the number of vaccine cellular components. For example, it has been shown that *E. coli* and ETEC can express a conserved N-Glycan heptasaccharide of *Campylobacter* and induce protective immunity against this pathogen [48,49]. The dose-sparing effect of the dmLT adjuvant also may help to reduce the number of cells needed per dose [50].

In addition, preclinical data from other studies suggest that inactivated whole-cell cholera vaccines, including vaccine strains engineered to also express other heterologous antigens, e.g., ETEC or *Helicobacter pylori,* could be evaluated/considered for inclusion in future combination vaccine options [51,52,53].

Our results show that the combination of multiple inactivated whole-cell strains of two important enteric pathogens is feasible for oral immunization without loss of immunogenicity. Further, co-administration with the safe and effective mucosal adjuvant dmLT improved the immune responses to both pathogens in the combination studied here and may be key to the future success of oral, inactivated whole-cell vaccines. Developing the combined or co-administration strategy to cover other enteric pathogens has the potential to be readily accomplished through maximizing expression of key, often conserved, antigens expressed on the bacterial cell surface. Since a major target population is young children in LMICs, oral enteric bacterial vaccines may have the benefit that, in addition to safely and effectively immunizing the target population directly, it may also be possible to use maternal immunization to help reduce the unacceptable impact these diseases now have on human health.

## Data Availability

The original contributions presented in this study are included in the article. Further inquiries can be directed to the corresponding author.

## References

[1] Khalil I.A. (2021). (Institute for Health Metrics and Evaluation, Seattle, WA, USA). Personal communication.

[2] Khalil I.A., Troeger C., Blacker B.F., Rao P.C., Brown A., Atherly D.E., Brewer T.G., Engmann C.M., Houpt E.R., Kang G. (2018). Morbidity and mortality due to shigella and enterotoxigenic *Escherichia coli* diarrhoea: The Global Burden of Disease Study 1990–2016. Lancet Infect. Dis..

[3] Troeger C., Blacker B.F., Khalil I.A., Rao P.C., Cao S., Zimsen S.R., Albertson S.B., Stanaway J.D., Deshpande A., Abebe Z. (2018). Diarrhoeal Disease Collaborators. Estimates of the global, regional, and national morbidity, mortality, and aetiologies of diarrhoea in 195 countries: A systematic analysis for the Global Burden of Disease Study 2016. Lancet Infect. Dis..

[4] Khalil I., Anderson J.D., Bagamian K.H., Baqar S., Giersing B., Hausdorff W.P., Marshall C., Porter C.K., Walker R.I., Bourgeois A.L. (2023). Vaccine value profile for enterotoxigenic Escherichia coli (ETEC). Vaccine.

[5] Hausdorff W.P., Anderson J.D.t., Bagamian K.H., Bourgeois A.L., Mills M., Sawe F., Scheele S., Talaat K., Giersing B.K. (2023). Vaccine value profile for Shigella. Vaccine.

[6] Riddle T.D., Cachafiero S.P., Putnam S.D., Hooper T.I. (2008). Development of a travelers’ diarrhea vaccine for the military: How much is an once of prevention really worth?. Vaccine.

[7] Olson S., Hall A., Riddle M.S., Porter C.K. (2019). Travelers’ diarrhea: Update on the incidence, etiology and risk in military and similar populations—1990–2005 versus 2005–2015, does a decade make a difference?. Trop. Dis. Travel Med. Vaccines.

[8] World Health Organization (2024). Diarrhoeal Disease Fact Sheet.

[9] PATH (2023). DefeatDD Report.

[10] Dhimal M., Bhandari D., Karki K.B., Shrestha S.L., Khanal M., Shrestha R.R.P., Dahal S., Bista B., Ebi K.L., Cisse G. (2022). Effects of Climatic Factors on Diarrheal Diseases among Children below 5 Years of Age at National and Subnational Levels in Nepal: An Ecological Study. Int. J. Environ. Res. Public Health.

[11] Dhimal M., Bhandari D. (2023). Climate change and imperatives to ascertain causes of infectious diarrhoea in low-income and middle-income countries. Lancet Glob. Health.

[12] Colston J.M., Zaitchik B.F., Badr H.S., Burnett E., Ali S.A., Rayamajhi A., Satter S.M., Eibach D., Krumkamp R., May J. (2022). Associations Between Eight Earth Observation-Derived Climate Variables and Enteropathogen Infection: An Independent Participant Data Meta-Analysis of Surveillance Studies With Broad Spectrum Nucleic Acid Diagnostics. Geohealth.

[13] Puett C., Anderson J.D.t., Bagamian K.H., Muhib F., Scheele S., Hausdorff W.P., Pecenka C. (2023). Projecting the long-term economic benefits of reducing Shigella-attributable linear growth faltering with a potential vaccine: A modelling study. Lancet Glob. Health.

[14] Guerrant R.L., Bolick D.T., Swann J.R. (2021). Modeling Enteropathy or Diarrhea with the Top Bacterial and Protozoal Pathogens: Differential Determinants of Outcomes. ACS Infect. Dis..

[15] Hausdorff W.P., Anderson J.D.t., Bourgeois A.L., Clifford A., Fleming J.A., Muhib F., Pecenka C., Puett C., Riddle M.S., Scheele S. (2024). Reassessing potential economic value and health impact of effective Shigella vaccines. Bull. World Health Organ..

[16] Hasso-Agopsowicz M., Sparrow E., Cameron A.M., Sati H., Srikantiah P., Gottlieb S., Bentsi-Enchill A., Le Doare K., Hamel M., Giersing B.K. (2024). The role of vaccines in reducing antimicrobial resistance: A review of potential impact of vaccines on AMR and insights across 16 vaccines and pathogens. Vaccine.

[17] (2024). UN AMR Declaration of Sept. https://www.who.int/news-room/events/detail/2024/09/26/default-calendar/un-general-assembly-high-level-meeting-on-antimicrobial-resistance-2024.

[18] Hausdorff W.P., Madhi S.A., Kang G., Kabore L., Tufet Bayona M., Giersing B.K. (2024). Facilitating the development of urgently required combination vaccines. Lancet Glob. Health.

[19] Walker R.I., Bourgeois A.L. (2023). Oral inactivated whole cell vaccine for mucosal immunization: ETVAX case study. Front. Immunol..

[20] Walker R.I., Clifford A. (2015). Recommendations regarding the development of combined enterotoxigenic Eschericha coli and Shigella vaccines for infants. Vaccine.

[21] Riddle M.S., Louis Bourgeois A., Clifford A., Jeon S., Giersing B.K., Jit M., Tufet Bayona M., Ovitt J., Hausdorff W.P. (2023). Challenges and opportunities in developing a Shigella-containing combination vaccine for children in low- and middle-income countries: Report of an expert convening. Vaccine.

[22] Walker R.I. (2025). Conserved antigens for enteric vaccines. Vaccine.

[23] Large K., Ruiz A.A., Slovenski I., Vieira M., Moon S. (2025). The 30-year evolution of oral cholera vaccines: A case study of a collaborative network alternative innovation model. PLoS Glob. Public Health.

[24] Clements J.D., Norton E.B. (2018). The Mucosal Vaccine Adjuvant LT(R192G/L211A) or dmLT. mSphere.

[25] Norton E.B., Lawson L.B., Freytag L.C., Clements J.D. (2011). Characterization of a mutant Escherichia coli heat-labile toxin, LT(R192G/L211A), as a safe and effective oral adjuvant. Clin. Vaccine Immunol..

[26] Qadri F., Akhtar M., Bhuiyan T.R., Chowdhury M.I., Ahmed T., Rafique T.A., Khan A., Rahman S.I.A., Khanam F., Lundgren A. (2020). Safety and immunogenicity of the oral, inactivated, enterotoxigenic Escherichia coli vaccine ETVAX in Bangladeshi children and infants: A double-blind, randomised, placebo-controlled phase 1/2 trial. Lancet Infect. Dis..

[27] Sukwa N., Mubanga C., Hatyoka L.M., Chilyabanyama O.N., Chibuye M., Mundia S., Munyinda M., Kamuti E., Siyambango M., Badiozzaman S. (2023). Safety, tolerability, and immunogenicity of an oral inactivated ETEC vaccine (ETVAX(R)) with dmLT adjuvant in healthy adults and children in Zambia: An age descending randomised, placebo-controlled trial. Vaccine.

[28] Hossain M.J., Svennerholm A.M., Carlin N., D’Alessandro U., Wierzba T.F. (2023). A Perspective on the Strategy for Advancing ETVAX((R)), An Anti-ETEC Diarrheal Disease Vaccine, into a Field Efficacy Trial in Gambian Children: Rationale, Challenges, Lessons Learned, and Future Directions. Microorganisms.

[29] Mubanga C., Simuyandi M., Mwape K., Chibesa K., Chisenga C., Chilyabanyama O.N., Randall A., Liang X., Glashoff R.H., Chilengi R. (2023). Use of an ETEC Proteome Microarray to Evaluate Cross-Reactivity of ETVAX((R)) Vaccine-Induced IgG Antibodies in Zambian Children. Vaccines.

[30] Kantele A., Riekkinen M., Jokiranta T.S., Pakkanen S.H., Pietila J.P., Patjas A., Eriksson M., Khawaja T., Klemets P., Marttinen K. (2023). Safety and immunogenicity of ETVAX(R), an oral inactivated vaccine against enterotoxigenic Escherichia coli diarrhoea: A double-blinded, randomized, placebo-controlled trial amongst Finnish travellers to Benin, West Africa. J. Travel Med..

[31] Kantele A., Carlin N., Jokiranta S., Svennerholm A.M. A phase 2b placebo controlled clinical trial of the oral vaccine ETVAX^®^ to examine safety, immunogenicity, diagnostic methods and protective efficacy against travelers’ diarrhea among Finnish travelers to Benin, West Africa. Proceedings of the 2022 VASE Virtual Symposium.

[32] (2024). Scandinavian Biopharma Announces Successful Completion of Pediatric Phase IIb Trial for ETVAX® Vaccine in The Gambia. https://scandinavianbiopharma.se/scandinavian-biopharma-announces-successful-completion-of-pediatric-phase-iib-trial-for-etvax-vaccine-in-the-gambia/.

[33] Akhtar M., Nizam N.N., Basher S.R., Hossain L., Akter S., Bhuiyan T.R., Qadri F., Lundgren A. (2021). dmLT Adjuvant Enhances Cytokine Responses to T Cell Stimuli, WholeCell Vaccine Antigens and Lipopolysaccharide in Both Adults and Infants. Front. Immunol..

[34] Svennerholm A.M., Lundgren A., Leach S., Akhtar M., Qadri F. (2021). Mucosal Immune Responses Against an Oral Enterotoxigenic Escherichia coli Vaccine Evaluated in Clinical Trials. J. Infect. Dis..

[35] Albert M.J., Haridas S., Ebenezer M., Raghupathy R., Khan I. (2015). Immunization with a Double-Mutant (R192G/L211A) of the Heat-Labile Enterotoxin of Escherichia coli Offers Partial Protection against Campylobacter jejuni in an Adult Mouse Intestinal Colonization Model. PLoS ONE.

[36] Albert M.J., Mustafa A.S., Islam A., Haridas S. (2013). Oral immunization with cholera toxin provides protection against Campylobacter jejuni in an adult mouse intestinal colonization model. mBio.

[37] Sandefur P.D., Peterson J.W. (1977). Neutralization of Salmonella toxin-induced elongation of Chinese hamster ovary cells by cholera antitoxin. Infect. Immun..

[38] Maier N., Grahek S.L., Halpern J., Restrepo S., Troncoso F., Shimko J., Torres O., Belkind-Gerson J., Sack D.A., Svennerholm A.M. (2023). Efficacy of an Enterotoxigenic Escherichia coli (ETEC) Vaccine on the Incidence and Severity of Traveler’s Diarrhea (TD): Evaluation of Alternative Endpoints and a TD Severity Score. Microorganisms.

[39] Kaminski R.W., Oaks E.V. (2009). Inactivated and subunit vaccines to prevent shigellosis. Expert Rev. Vaccines.

[40] McKenzie R., Walker R.I., Nabors G.S., Van De Verg L.L., Carpenter C., Gomes G., Forbes E., Tian J.H., Yang H.H., Pace J.L. (2006). Safety and immunogenicity of an oral, inactivated, whole-cell vaccine for Shigella sonnei: Preclinical studies and a Phase I trial. Vaccine.

[41] Chakraborty S., Harro C., DeNearing B., Bream J., Bauers N., Dally L., Flores J., Van de Verg L., Sack D.A., Walker R. (2016). Evaluation of the Safety, Tolerability, and Immunogenicity of an Oral, Inactivated Whole-Cell Shigella flexneri 2a Vaccine in Healthy Adult Subjects. Clin. Vaccine Immunol..

[42] Holmgren J., Bourgeois L., Carlin N., Clements J., Gustafsson B., Lundgren A., Nygren E., Tobias J., Walker R., Svennerholm A.M. (2013). Development and preclinical evaluation of safety and immunogenicity of an oral ETEC vaccine containing inactivated E. coli bacteria overexpressing colonization factors CFA/I, CS3, CS5 and CS6 combined with a hybrid LT/CT B subunit antigen, administered alone and together with dmLT adjuvant. Vaccine.

[43] Kaminski R.W., Wu M., Turbyfill K.R., Clarkson K., Tai B., Bourgeois A.L., Van De Verg L.L., Walker R.I., Oaks E.V. (2014). Development and preclinical evaluation of a trivalent, formalin-inactivated Shigella whole-cell vaccine. Clin. Vaccine Immunol..

[44] Villavedra M., Carol H., Hjulstrom M., Holmgren J., Czerkinsky C. (1997). “PERFEXT”: A direct method for quantitative assessment of cytokine production in vivo at the local level. Res. Immunol..

[45] Johansson E.L., Rask C., Fredriksson M., Eriksson K., Czerkinsky C., Holmgren J. (1998). Antibodies and antibody-secreting cells in the female genital tract after vaginal or intranasal immunization with cholera toxin B subunit or conjugates. Infect. Immun..

[46] Kim M.J., Moon Y.H., Kim H., Rho S., Shin Y.K., Song M., Walker R., Czerkinsky C., Kim D.W., Kim J.O. (2018). Cross-Protective Shigella Whole-Cell Vaccine With a Truncated O-Polysaccharide Chain. Front. Microbiol..

[47] Fleming J.A., Gurley N., Knudson S., Kabore L., Bawa J.T., Dapaah P., Kumar S., Uranw S., Tran T., Mai L.T.P. (2023). Exploring Shigella vaccine priorities and preferences: Results from a mixed-methods study in low- and middle-income settings. Vaccine X.

[48] Nothaft H., Bian X., Shajahan A., Miller W.G., Bolick D.T., Guerrant R.L., Azadi P., Ng K.K.S., Szymanski C.M. (2021). Detecting Glucose Fluctuations in the Campylobacter jejuni N-Glycan Structure. ACS Chem. Biol..

[49] Nothaft H., Perez-Munoz M.E., Yang T., Murugan A.V.M., Miller M., Kolarich D., Plastow G.S., Walter J., Szymanski C.M. (2021). Improving Chicken Responses to Glycoconjugate Vaccination Against Campylobacter jejuni. Front. Microbiol..

[50] Qadri F., Wierzba T.F., Ali M., Chowdhury F., Khan A.I., Saha A., Khan I.A., Asaduzzaman M., Akter A., Khan A. (2016). Efficacy of a Single-Dose, Inactivated Oral Cholera Vaccine in Bangladesh. N. Engl. J. Med..

[51] Sharma T., Joshi N., Kumar Mandyal A., Nordqvist S.L., Lebens M., Kanchan V., Löfstrand M., Jeverstam F., Mainul Ahasan M., Khan I. (2020). Development of Hillchol^®^, a low-cost inactivated single strain Hikojima oral cholera vaccine. Vaccine.

[52] Tobias J., Svennerholm A.M. (2012). Strategies to overexpress enterotoxigenic Escherichia coli (ETEC) colonization factors for the construction of oral whole-cell inactivated ETEC vaccine candidates. Appl. Microbiol. Biotechnol..

[53] Tobias J., Svennerholm A.M., Holmgren J., Lebens M. (2010). Construction and expression of immunogenic hybrid enterotoxigenic Escherichia coli CFA/I and CS2 colonization fimbriae for use in vaccines. Appl. Microbiol. Biotechnol..

